# Understanding barriers and facilitators to participation in Parkinson’s research in Black communities in the UK

**DOI:** 10.1177/1877718X251351990

**Published:** 2025-06-20

**Authors:** Ivelina Dobreva, Sabrina Kalam, Moïse Roche, Ece Bayram, Rimona S Weil, Angeliki Zarkali

**Affiliations:** Dementia Research Centre, Queen Square Institute of Neurology, University College London, London, UK; Department of Neurodegenerative Disease, 2nd Floor Russell Square House, 10-12 Russell Square, London WC1B 5EH, UK.; Dementia Research Centre, Queen Square Institute of Neurology, University College London, London, UK; Division of Psychiatry, University College London, London, UK; Department of Neurology, Movement Disorders Center, University of Colorado Anschutz, Aurora, CO, USA; Dementia Research Centre, Queen Square Institute of Neurology, University College London, London, UK; Movement Disorders Centre, University College London, London, UK; National Hospital for Neurology & Neurosurgery, London, UK; Dementia Research Centre, Queen Square Institute of Neurology, University College London, London, UK; National Hospital for Neurology & Neurosurgery, London, UK

**Keywords:** Parkinson’s disease, ethnicity, ethnic and racial minorities, patient participation

## Abstract

People from Black backgrounds are underrepresented in Parkinson’s research, despite evidence of higher disease burden and risk of dementia. Greater understanding of the factors influencing participation in Parkinson’s research can improve recruitment, quality and generalizability of both observational research studies and clinical trials. Through focus groups with 17 people with Parkinson’s and carers from Black communities, we identified distrust in the research process, stigma of Parkinson’s diagnosis, and accessibility as key barriers to research participation. Participants made recommendations including: raising awareness of Parkinson’s and related research, involving community ambassadors, improving communication throughout the research process, and providing practical support.

## Plain language summary

People with Parkinson’s from Black communities often face delays in diagnosis. They are also more likely to experience severe symptoms and are at higher risk of dementia. However, they are not well represented in Parkinson’s research. This means that research studies do not fully represent their experiences or needs, and that outcomes from research may not be fully relevant to them. In this project, we aimed to understand the reasons Black people may choose not to participate in Parkinson’s research and how researchers can encourage participation. We recruited 17 people with Parkinson’s and their carers from Black communities to participate in focus groups. We asked them what they think about participating in research; to share previous experiences (positive and negative); and what would make taking part in Parkinson’s research easier for them. We found that the main reasons people from Black backgrounds do not participate in research are distrust, stigma from Parkinson’s, and how convenient it is to participate. Despite this, they still would like to help and join research projects. Participants suggested ways to make research more inclusive, such as: sharing information about Parkinson’s research within Black communities; working with trusted community members to spread the word; keeping participants informed throughout the research process; and offering practical support such as transport arrangements on the day of research.

Much of our understanding of Parkinson’s disease (PD) is based on research disproportionally conducted in populations of European ancestry.^1^ Inclusion of people from Black African and Black Caribbean ethnicity remains disproportionately low.^1-3^

Including diverse populations is particularly important in PD research due to the well-recognized heterogeneity in clinical presentation and progression. Recent evidence suggests phenotypic and genetic differences are present between ethnic groups. Black people with Parkinson’s (PwP) are at a higher-risk of cognitive decline, dementia, and mortality compared to White PwP.^3,4^ Comorbidities such as small vessel disease, more prevalent in Black populations, may exacerbate poor outcomes by accelerating neurodegenerative processes.^5,6^ Recent work in African populations identified a novel branchpoint sequence alteration in the GBA1 gene^7^ with a different functional mechanism than European populations– associated with increased PD risk and earlier onset.^8^ This suggests disease pathways may vary across ethnic groups.

We urgently require an increase in research engagement from different ethnic groups to better understand the underlying causes of PD and its progression. We undertook a research enrichment initiative to explore the factors affecting participation in PD research in Black populations living in UK.

In total, 17 people (10 PwP, 7 carers) were recruited from a database of PwP who had either taken part in previous projects from our group, or expressed an interest in research at our institution; or through a UK PD charity (Parkinson’s UK). During four focus groups, open-ended questions were used to explore attitudes towards research and willingness to participate in research projects, and previous experience of research participation (both positive and negative). Each focus group lasted approximately 1h30 min led by two facilitators (AZ, MR) and two notetakers (ID, SK). Interview questions, informed by stakeholder input, are presented in Table 1A. During the focus groups, discussions and comments were transcribed (ID, SK); and responses were recorded without personal or identifiable information. The focus group transcripts were collated and analyzed using an inductive thematic analysis.^9^ Two researchers (ID and SK) independently developed codes using NVivo (v14.23.2) through an iterative method, with multiple reading of the raw notes and recoding of the data until no further codes emerged. Coded data were reviewed, and similar categories were discussed until consensus themes were constructed.

Participants described both broader societal benefits of participating in research, such as scientific discovery and benefiting future generations, and personal benefits, such as to gain insights into managing their own symptoms. Despite this, the main focus of research was perceived to test new treatments, and as such to be “a gamble”, “scary” and “risky”.

We conceptualized 3 themes relating to barriers to participating in Parkinson’s research: (1) distrust in medical and scientific institutions, (2) stigma of PD diagnosis; and (3) accessibility and convenience.

Distrust was associated with perceived lack of transparency and the belief that research primarily benefits research institutions or other populations, rather than Black communities. This distrust related to historical knowledge of intentional mistreatment, exposure to unnecessary risks, and being subjected to experimentation, described by participants as being treated like a “lab rat” or “guinea pig”.

A second barrier to research participation was the risk of inadvertently disclosing a PD diagnosis to family or friends by taking part in research, leading to concerns about potential stigma. Participants expressed a desire to keep diagnosis private and were reluctant to share details about their condition. Stigma was tightly linked to a perceived lack of PD awareness amongst Black communities, with one participant explaining that PD is perceived as “not a Black disease”.

Finally, inconvenience and lack of accessibility to attend research visits, particularly in relation to challenging PD symptoms and medication schedules was raised. Considerations such as time of day, burden of travel to a research venue, fluctuations in motor and non-motor symptoms, and medication side effects were all discussed.

Despite these barriers, participants were generally willing to engage with research if certain expectations were met. We report four main recommendations that arose from our focus groups as potential aids to facilitate research participation (Table 1B).

A key suggestion was to raise awareness of PD and of scientific research into the condition, among Black communities. For example, including family members and carers in research where possible, highlighting ongoing PD research on formal and social media platforms, and encouraging those who have already participated to share their experience with their communities directly. Institutional reputation was discussed, with participants more likely to trust a project conducted or endorsed from large, well-recognized organizations or institutions.

Community engagement strategies specifically designed to educate and raise PD awareness are known to increase recruitment of underrepresented groups in PD research. A recent project successfully utilized educational activities to boost recruitment of underrepresented groups for an online PD study.^2^

Such initiatives may put pressure on projects with limited funding or short-term timelines. One scalable strategy is to implement a community ambassador program, partnering with individuals who already have close relationships with target communities. Such strategies have successfully increased engagement with clinical trials in Black and Latino communities in the US.^10^

Having an ambassador who can represent the community was also raised as a motivator for people to engage with research in our focus groups. For example, a high-profile celebrity from the community, diagnosed with Parkinson’s, could have the potential to change people’s perspective of PD and “*set an example, by showing resilience, continuing to live their life, and representing the community*”. Importantly, ambassadors were recognised to establish a sense of trust towards health research and bring awareness of PD as “not just a White’s disease”.

Regarding practical recommendations, sharing educational resources with research participants was suggested. For example, participants being given information on health status where available, access to information about living with PD, symptom management and lifestyle advice, as well as gaining access to relevant events, such as seminars and talks about PD were suggested.

Participants requested consistent communication through various channels (e.g., email, phone, text) depending on the PwP’s needs. They highlighted the importance of using appropriate style and framing, avoiding language that directly emphasizes ethnicity as the reason for an individual’s invitation to participate. Such messaging can feel tokenistic or alienating, potentially reinforcing feelings of being singled-out rather than genuinely included. PwP advised researchers to “not be afraid to invite all people to participate” and highlight the importance of diversity in research and need for representation of different communities, without targeting individuals solely based on their ethnicity. For instance, instead of directly stating, “We invite Black participants,” the message could emphasize, “Help us ensure diversity in Parkinson’s research so that all communities are represented in our studies”.

More practical recommendations such as financial incentives, reimbursement for travel, offering refreshments and lunch, and being flexible about timings of research visits, were all seen as means of facilitating research uptake.

Financial incentives increase research participation in clinical trials,^11^ but may disproportionately influence lower socioeconomic groups who may rely on such incentives, raising ethical concerns. Further, financial incentives may not be feasible for all research institutions. In such cases, other incentives such as participation timing, or light refreshment during study visits can be useful.^12^ Further, while flexible scheduling and travel reimbursements can support participation, their effectiveness may be context dependent. For example, in communities with limited access to transportation or constrained work schedules, other means of support may be more useful.

The findings from our focus groups present opportunities for researchers and allied professionals to develop novel engagement strategies and amend existing research protocols to improve uptake of under-represented groups. We offer pragmatic, easy to implement and straightforward suggestions, directly informed by discussions with Black PwP. Implementing these strategies could not only improve research engagement but also help reduce healthcare disparities and ensure that future research is more inclusive and representative.

Our study was limited to individuals from Black communities residing in the UK who have expressed an interest in research. Thus, our participants may be a more self-reflective group and more comfortable engaging with research studies, impacting their responses. Cultural beliefs and attitudes toward research may vary significantly across different countries and contexts, so findings may not be generalizable to Black and other minoritized populations in other regions. As this work was completed as a research enrichment initiative, the findings provide preliminary insights that warrant further investigation with larger, more representative samples.

## Figures and Tables

**Table 1.** (A) Semi-structured interview questions.

What does (the word) ‘research’ mean to you?		
What does a research visit mean to you?		
What is your experience of research?		
How do you find participating in research?		
- Prompt: do you typically find it easy, challenging or any other way?		
What would encourage or help you to participate in research?		
What would make it difficult or challenging for you to participate in research?		
How can researchers make it easier for people from Black communities to engage with PD research (Thinking about previous experience of research)?		
What did you like in research you previously took part in?		
What did you dislike, or made you think, this could have been done better?		
What would have made it better?		
(B) Recommendations for increasing research participation in Parkinson’s (PD) research from Black communities.		
Theme	Mitigation strategies	Selected quotes
Increase awareness of Parkinson’s and research in Black communities	Educate the Black community about Parkinson’s; Highlight ongoing research during patient/peer support meetings, or on media outlets; Encourage people with Parkinson’s to share personal experience of participating in research to local community groups.	“*People of colour are not aware of Parkinson’s – they consider Parkinson’s not to be a Black disease*.” “*We need education of the public and raising awareness of Parkinson’s disease amongst minorities*.” “*Media coverage of Parkinson’s would help in awareness and bringing people to research*.” “*It would be good to see how that friend is getting along before I decide to participate in research*.” “*It helps to put information out there and show how things are tested. It would help over time the degree of acceptance*.”
Ambassadors / role models	A high-profile person of colour representing the community and advertising research into Parkinson’s disease; Setting an example of living well with Parkinson’s	“*Celebrities of colour who have the disease and manage to live with it is a fantastic source of awareness. [They can be] a figure which can change the perspective of the condition – someone who has the condition and still lives their life as a normal person*.” “*The point [of an ambassador] is that the person has the condition, but they show resilience*” “*Having high profile people of colour with Parkinson’s speaking up and showing that research is safe – I would trust them*.”
Effective communication and feedback	Offer individual feedback or information on the participant’s health where possible; Provide access to educational resources on Parkinson’s even if not directly related to the topic of the research; Share appropriate information on study aims and purpose; Maintain regular communication from the team regarding group-level findings including sharing resulting publications; Shift focus away from ethnicity during recruitment; State clearly the institution conducting the research.	“*Provide information about the condition when participating in research would be another incentive; – It would be nice to know results from scans or cognition tests, I understand that it’s research, but it would be good to be told if there are any relevant clinical findings – e.g if they found diabetes*.” “*Educational resources being provided as part of the project would help; for example, leaflets, case studies, advice on ways to cope with the condition, suggested dietary changes, if any foods can trigger reactions in people with PD – e.g., avoid these foods, try these foods*” “*Too much talk on your ethnicity, e.g., As an ethnic person, come to this research – makes you feel uncomfortable*.”
Practical support and convenience of participation	Offer flexibility regarding timing of attendance; Make transport arrangements and/or offer travel reimbursement; Provide financial incentives and refreshments where possible; Invite family members for collateral information and support on the day.	“*How close the research is and when to go during the day e.g., afternoon or morning (I can be stiff in the morning until my meds kick in)*” “*What’s in it for me during participation [is important]– financial benefit, or a lunch/breakfast, a good chat*.”

## Data Availability

The data supporting the findings of this study are available from the corresponding author, upon reasonable request.
